# Emilin1 gene and essential hypertension: a two-stage association study in northern Han Chinese population

**DOI:** 10.1186/1471-2350-10-118

**Published:** 2009-11-18

**Authors:** Chong Shen, Xiangfeng Lu, Yun Li, Qi Zhao, Xiaoli Liu, Liping Hou, Laiyuan Wang, Shufeng Chen, Jianfeng Huang, Dongfeng Gu

**Affiliations:** 1 Department of Evidence Based Medicine and Division of Population Genetics, Cardiovascular Institute and Fuwai Hospital, Chinese Academy of Medical Sciences and Peking Union Medical College P eople's Republic of China, No. 167 Beilishi Rd, Beijing 100037, PR China; 2 Department of Epidemiology and Biostatistics, School of Public Health, Nanjing Medical University, Nanjing 210029, PR China

## Abstract

**Background:**

Elastogenesis of elastic extracellular matrix (ECM) which was recognized as a major component of blood vessels has been believed for a long time to play only a passive role in the dynamic vascular changes of typical hypertension. Emilin1 gene participated in the transcription of ECM's formation and was recognized to modulate links TGF-β maturation to blood pressure homeostasis in animal study. Recently relevant advances urge further researches to investigate the role of Emilin1 gene in regulating TGF-β signals involved in elastogenesis and vascular cell defects of essential hypertension (EH).

**Methods:**

We designed a two-stage case-control study and selected three single nucleotide polymorphisms (SNPs), rs3754734, rs2011616 and rs2304682 from the HapMap database, which covered Emilin1 gene. Totally 2,586 subjects were recruited from the International Collaborative Study of Cardiovascular Disease in Asia (InterASIA). In stage 1, all the three SNPs of the Emilin1 gene were genotyped and tested within a subsample including 503 cases and 490 controls, significant SNPs would enter into stage 2 including 814 cases with hypertension and 779 controls and analyze on the basis of testing total 2,586 subjects.

**Results:**

In stage 1, single locus analyses showed that SNPs rs3754734 and rs2011616 had significant association with EH (P < 0.05). In stage 2, weak association for dominant model were observed by age stratification and odds ratio (ORs) of TG+GG vs. TT of rs3754734 were 0.768 (0.584-1.009), 0.985 (0.735-1.320) and 1.346 (1.003-1.806) in < 50, 50-59 and ≥ 60 years group and ORs of GA+AA vs. GG of rs2011616 were 0.745 (0.568-0.977), 1.013 (0.758-1.353) and 1.437 (1.072-1.926) in < 50, 50-59 and ≥ 60 years group respectively. Accordingly, significant interactions were detected between genotypes of rs3754734 and rs2011616 and age for EH, and ORs were 1.758 (1.180-2.620), P = 0.006 and 1.903 (1.281-2.825), P = 0.001, respectively. Results of haplotypes analysis showed that there weren't any haplotypes associated with EH directly, but the interaction of hap2 (GA) and age-group found to be significant after being adjusted for the covariates, OR was 1.220 (1.031-1.444), P value was 0.020.

**Conclusion:**

Our findings don't support positive association of Emilin1 gene with EH, but the interaction of age and genotype variation of rs3754734 and rs2011616 might increase the risk to hypertension.

## Background

As a major health problem affecting about one third of the adult worldwide, hypertension mostly arises as a complex quantitative trait that is affected by varying combinations of genetic and environmental factors [1,2]. In the majority of cases, the development of high blood pressure is idiopathic being termed essential hypertension[3]. Recently, studies have highlighted new roles of resistance artery narrowing and large artery stiffening recognized as hallmarks of essential hypertension, which increase peripheral resistance and compromise vascular compliance, respectively [4-6].

A series of studies on the pathogenesis of hypertension have placed major emphasis on smooth muscle and endothelial cells[4], which are in continuous cross-talk with each other and formulate dynamic structures of blood vessels. Remarkably, Elastogenesis of elastic extracellular matrix (ECM), another major component of blood vessels, has been considered for a long time to play only a passive role in the dynamic vascular changes of typical hypertension [2,4,7-9]. Interactions of ECM-cell named as elastic fibres, together with neurotransmitters and hormones modulate the structural organization of the vascular wall and provide the structural framework and physiological circulatory function through specific receptors [2]. Therefore, dysfunction of elastic fibres might be key elements in the pathophysiological changes of hypertensive vascular remodeling.

EMILIN, which was originally identified in attempts to isolate ECM, was named for elastin micro fibril interface located protein for its peculiarly fine distribution on the surface of amorphous elastin [10]. As a main member of EMILIN, Emilin1 might play a key role in hypertensive vascular remodeling [7,9,10]. Transforming growth factor (TGF)-β proteins are main regulators of blood vessel development and maintenance, and Emilin1 inhibits TGF-β signaling by binding specifically to the proTGF-β precursor and preventing its maturation by furin convertases in the extracellular space [2,8]. Therefore, based on the proof of Emilin1 modulating TGF-β availability in the development of cardiovascular system and the pathogenesis of hypertension and linking TGF-β maturation to blood pressure homeostasis identified in animal study, the discovery of genetic susceptibility of Emilin1 gene to hypertension will lead to a better understanding of the mechanism of human hypertension.

In the present study, we conducted a two-stage case-control study [11] to investigate the associations of common variants of Emilin1 gene with EH in the northern Han Chinese population.

## Methods

### Subjects

All the studied subjects were recruited from the International Collaborative Study of Cardiovascular Disease in Asia (InterASIA in China), from which all the DNA samples and clinical data for participants were obtained [12]. The local bioethical committee approved the protocol, and informed consent was obtained from each participant. InterASIA used a four-stage stratified sampling method to select a nationally representative sample of the general population aged 35 to 74 years in China [12,13]. A total of 15,838 persons accepted the survey and examination. Among these, we enrolled 1,317 unrelated hypertensive patients and 1,269 age and gender-matched unrelated normotensives from four northern field centers of InterASIA, namely Beijing, Jilin, Shandong, and Shanxi province. Three BP measurements were obtained from each participant by certified observers according to a standard protocol recommended by the American Heart Association [14,15].

In this study, hypertensives were selected from those with systolic BP (SBP) ≥ 150 mmHg, and/or diastolic BP (DBP) ≥ 95 mmHg, or currently administering antihypertensive medication. Subjects with a clinical history of secondary hypertension, coronary heart disease or diabetes were excluded. At the same time, normotensives with SBP < 140 mmHg and DBP < 90 mmHg were selected from the same target study population as controls.

A two-stage association study of total 2,586 unrelated subjects as main study population was conducted [16]. Cases with high BP were selected likely to enrich genetic susceptibility [17] by increasing the difference in frequencies of susceptibility alleles between cases and controls to improve statistical power. 993 subjects were selected as stage 1 subsample including 503 hypertensive patients (SBP ≥ 160 mmHg and/or DBP ≥ 100 mmHg) and 490 age- and gender-matched normotensives (SBP < 140 mmHg and DBP < 90 mmHg). In stage 2, subsample included 814 cases with hypertension and 779 age- and gender-matched normotensives. Additionally, previous genomic control analysis revealed no evidence of population stratification with this data in previous study [18].

### Single nucleotide polymorphisms (SNP) selecting and genotyping

Emilin1 gene (Official Symbol: EMILIN1, Gene ID: 11117) was mapped on chromosome 2 p23 and spanned 732, 9 bp with 8 entries exons. We searched the HapMap data of Han Chinese in Beijing, China (CHB) (http://www.hapmap.org/, public release up to Mar 2008), and selected all SNPs from 5 kb upstream of Emilin1 gene to 1 kb downstream. Three SNPs, rs3754734 (-1932G/T) at 5' flanking region, rs2011616 (1031G/A) in intron_1 and rs2304682 (6239 G/C) in intron 5, were identified. SNP rs3754734 (-1932G/T) may predict buildups of transcription factor (TF) binding sites of Nkx-2 and CdxA according to the variation of allele G to T. Function analysis proceeded on the website of selection tool for single nucleotide polymorphisms (FASTSNP, http://fastsnp.ibms.sinica.edu.tw) [19] and the risk of rs3754734 for TF binding sites is 1-3. Also, TF binding site of stimulating protein 1 (Sp1) was predicted as an intronic enhancer for allele G to allele A of rs2011616 and risk is 1-2.

Blood for genotyping was taken into ethylenediamine tetraacetic acid (EDTA)-containing receptacles. All the 3 entries SNPs were genotyped by polymerase chain reaction/restriction fragment length polymorphism (PCR/RFLP) protocols. The primers, locations for Emilin1 gene, alleles, minor allele frequency (MAF), related restriction endonuclease, as well as digested bands are shown in table 1. Digested fragments were separated by electrophoresis in 3% agarose gel and identified by ethidium bromide staining. Ninety-six randomly selected individuals were genotyped again for quality control with complete concordance and genotyping was done blindly to the case-control status in Chinese National Human Genome Center, Beijing.

**Table 1:** Location region, primer sequences, alleles, MAF and restriction enzymes for Emilin1 gene SNPs

SNPs	Emilin1 region	Primer sequences	Alleles Major/Minor	MAF	Restriction enzymes	Digested bands (bp)
Rs3754734	5'fanking	F: 5' CGCTCTCACCCACAATACCT 3' R: 5' TGGCAAATGGTAAATGCTCA 3'	T/G	0.367	Apo I	T allele: 191 G allele: 159
Rs2011616	Intron 1	F: 5' TTGACCCCATCCAGTAATC 3' R: 5'AATAAGCCATTGATGAGCCTGAC 3'	G/A	0.356	Hinf I	G allele: 153 A allele: 129
Rs2304682	Intron 5	F: 5' TGCTTTGAAGTCCACGTAGCATC 3' R: 5' TGGGAAGGGTGGATGTTA 3'	G/C	0.367	Taq I	G allele: 256 C allele: 279

Underlined bases were mismatched

F: forward primer, R: reverse primer

MAF: Minor allele frequency.

### Statistical Analysis

Unpaired Student's t-test was used to test the differences of all measured variables presented as the means ± SD between cases and controls, and one-way ANOVA was used to test differences in SBP and DBP among genotypes.

In stage 1, single locus analyses were used to test the associations between three SNPs and EH. The numbers of alleles and genotypes of the SNPs were counted, and their distributions between the case and control groups were compared by Chi-square (χ^2^) test. Hardy-Weinberg equilibrium (HWE) test were used to detect genotype typing errors for quality control by Fisher's exact χ^2 ^test using the program HWE [20]. A forward stepwise (likelihood ratio) method for multivariate unconditional logistic regression was applied to adjust for covariates including age, gender, body mass index (BMI), glucose (Glu), triglycerides (TG), total cholesterol (TC), high density lipoprotein cholesterol (HDL-C), low density lipoprotein cholesterol (LDL-C), creatinine (Cr), smoking and drinking status. The probability for stepwise entry is 0.05 and that for stepwise removal is 0.1. The criterion for SNPs entering into stage 2 study was that the SNPs had a two-tailed probability (P) value of < 0.05 for the comparisons of allele frequencies or genotype distributions between cases and controls in stage 1. Bonferroni correction for multiple comparisons wasn't used in stage 1 because stage 1 served for hypothesis generation. In stage 2, joint analysis was performed in the main study population for the high efficiency and power reliability because the power of detecting significant association is reduced by using only the samples in stage 2 [21,22]. Particularly, gene-age interaction was analyzed with age stratification under 50 years, 50-60 years and over 60 years.

Multilocus analyses for haplotype (frequency > 5%) were performed in stage 2. The haplo.glm approach was used [23] to obtain ORs of risk haplotypes in the multivariable-adjusted model. Haplo.glm was applied in the haplo.stats package using R language http://cran.r-project.org/.

## Results

Table 2 shows the demographic and clinical characteristics of the subjects included in the stage 1 and stage 2 and joint population. Age and the percentage of men, drinking and smoking were not significantly different between cases and controls in both stage 1 and stage 2. The cases generally had lower HDL-C and higher BMI, TC, TG, Glu and Cr levels than the corresponding controls. As expected, the SBP and DBP levels were both significantly higher in the cases of stage 1 than those of stage 2 (P values < 0.0001).

**Table 2:** Comparison of clinical characteristics between cases and controls in stage 1, stage 2 and joint analysis

	Stage 1		Stage 2		Joint analysis	
			
Characteristics	Cases (n = 503)	Controls (n = 490)	Cases (n = 814)	Controls (n = 779)	Cases (n = 1317)	Controls (n = 1269)
Male (%)	52.1	52.4	48.3	51.5	49.7	51.9
Age (years)	53.6 ± 9.3	53.5 ± 9.2	54.5 ± 10.7	53.5 ± 9.7	54.2 ± 10.2	53.5 ± 9.5
SBP (mmHg)	177.07 ± 28.05*⊿	117.47 ± 11.64⊿	148.98 ± 17.84*	113.81 ± 9.86	159.70 ± 26.13*	115.21 ± 10.72
DBP (mmHg)	104.34 ± 12.28*⊿	75.05 ± 8.00⊿	90.62 ± 9.81*	72.86 ± 7.57	95.85 ± 12.70*	73.69 ± 7.81
BMI (kg/m2)	26.32 ± 3.85*	24.30 ± 3.56	25.79 ± 3.52*	23.85 ± 3.37	26.00 ± 3.67*	24.03 ± 3.43
TC (mg/dl)	198.64 ± 37.50****	192.46 ± 40.06	197.34 ± 35.82*	188.43 ± 38.15	197.84 ± 36.46*	189.98 ± 38.93
HDL-C (mg/dl)	48.80 ± 11.83***	51.31 ± 13.07	48.38 ± 11.14*	49.39 ± 11.43	48.54 ± 11.41**	50.13 ± 12.12
LDL-C (mg/dl)	121.28 ± 32.64****	117.38 ± 32.98	122.08 ± 32.69*	115.81 ± 34.36	121.78 ± 32.66*	116.42 ± 33.83
TG (mg/dl)	150.58 ± 94.21*	126.62 ± 76.42	134.94 ± 65.09*	116.67 ± 55.37	140.92 ± 77.85*	120.51 ± 64.47
Glu (mg/dl)	106.77 ± 32.40*	99.09 ± 18.77	97.28 ± 10.90*	94.80 ± 11.55	100.90 ± 22.25*	96.46 ± 14.91
Cr (μmol/l)	70.83 ± 15.21****	68.92 ± 12.12	70.92 ± 13.34	71.03 ± 12.93	70.89 ± 14.08	70.21 ± 12.66
Smokers (%)	40.6	43.1	39.7	43.0	40.0	43.0
Drinkers (%)	34.4	33.5	30.1	28.4	31.7	30.3
Anti-hypertensive medication (%)	33.00		35.14		34.32	

*P < 0.0001 for comparison with corresponding controls

**P < 0.001 for comparison with corresponding controls

***P < 0.01 for comparison with corresponding controls

****P < 0.05 for comparison with corresponding controls

⊿ P < 0.0001 for comparison with corresponding variables in stage 2

Genotype distributions and allele frequencies for the three SNPs tested are showed in table 3. All SNPs were in HWE in cases, controls and combination population. There were significant differences in rs3754734 and rs2011616 genotypes distributions between cases and controls. Compared with wild-type individuals, both G allele carriers (TG/GG genotype)of rs3754734 and A allele carriers (GA/AA genotype)of rs2011616 were at increased risk for EH, ORs were 1.336(1.013-1.762) and 1.348 (1.035-1.756), respectively. There weren't significant differences in genotype or allele frequencies of rs2304682 between cases and controls. Therefore, rs3754734 and rs2011616 would be taken as further hypotheses to test and proceed with the stage 2 study.

**Table 3:** Genotype distributions and allele frequencies of the three SNPs tested in stage 1 and stage 2

SNP		Group	Genotype				Allele		
				
			WT	Ht+MT	OR^a ^(95%CI)	**P**^a^	Major/Minor	OR (95%CI)	P
Stage 1	rs3754734		TT	TG+GG			T/G		
		Case	153	261+89			0.564/0.436		
		Control	188	214+88	1.336(1.013-1.762)	0.040	0.602/0.398	1.17(0.98-1.40)	0.082
	rs2011616		GG	GA+AA			G/A		
		Case	244	214+43			0.701/0.299		
		Control	270	181+39	1.348(1.035-1.756)	0.027	0.736/0.264	1.19(0.98-1.45)	0.082
	rs2304682		GG	GC+CC			G/C		
		Case	245	213+45			0.699/0.301		
		Control	251	196+43	1.034(0.796-1.343)	0.840	0.712/0.288	1.07(0.88-1.29)	0.511
Stage 2	rs3754734		TT	TG+GG			T/G		
		Case	522	598+164			0.648/0.352		
		Control	533	570+163	0.980(0.832-1.155)	0.811	0.646/0.354	0.992(0.885-1.112)	0.894
	rs2011616		GG	GA+AA			G/A		
		Case	643	555+109			0.704/0.296		
		Control	608	541+110	1.004(0.853-1.181)	0.963	0.698/0.302	1.032 (0.915-1.163)	0.611

a. Adjusted for gender, age, BMI, TC, TG, Cr, HDL-C, LDL-C, Glu, smoking, drinking.

WT wide type, Ht heterozygote, MT mutant type

In stage 2, rs3754734 and rs2011616 were genotyped on the remaining cases and controls and conjoint analysis was conducted in all 2,586 individuals described as above. Genotype analysis was not found statistical association of rs3754734 and rs2011616 with EH after adjusted for gender, age, BMI, TC, TG, Cr, HDL-C, LDL-C, Glu, smoking, drinking (table 3).

In order to evaluate genetic modification effective of age for Emilin1 gene to EH, subjects were divided into < 50, 50-59 and ≥ 60 years groups in stage 2. The dominant models (TG+GG vs. TT) of rs3754734 showed negative correlation to EH in ≥ 60 years group and ORs was 1.346 (1.003-1.806). Compared with GG genotype, GA or AA genotype of rs2011616 showed a bidirectional effects on EH in < 50 and ≥ 60 years groups and ORs were 0.745 (0.568-0.977) and 1.437 (1.072-1.926) respectively (table 4). But there wasn't any statistical difference of allele distribution found between case and control among age groups. Accordingly, Logistic regression model (the entry probability value for stepwise 0.05 and the removal is 0.1) was used to detect significant interactions between genotypes of rs3754734 and rs2011616 and age for EH, and ORs were 1.758 (1.180-2.620), P = 0.006 and 1.903(1.281-2.825), P = 0.001, respectively (table 5).

**Table 4:** Analysis of age stratified association of rs3754734 and rs2011616 with hypertension in stage 2.

SNP	Age group (years)	Genotype				Allele (Major/Minor)			
			
		Case	Control	OR*(95%CI)	P	case	control	OR (95%CI)	P
rs3754734		TT/TG/GG		TT vs. TG/GG		T/G	T/G		
	< 50	217/193/58	188/220/59	0.768(0.584-1.009)	0.058	0.670/0.330	0.638/0.362	0.87(0.72-1.06)	0.174
	50-60	161/183/48	172/191/55	0.985(0.735-1.320)	0.919	0.644/0.356	0.640/0.360	1.02(0.83-1.25)	0.876
	> 60	175/222/58	173/159/49	1.346(1.003-1.806)	0.048	0.629/0.371	0.663/0.337	0.86(0.70-1.05)	0.151
rs2011616		GG/GA/AA		GG vs. GA/AA		G/A	G/A		
	< 50	245/177/41	212/213/38	0.745(0.568-0.977)	0.033	0.720/0.280	0.688/0.312	1.17(0.96-1.43)	0.126
	50-60	182/170/40	195/187/33	1.013(0.758-1.353)	0.932	0.681/0.319	0.695/0.305	0.94(0.76-1.16)	0.543
	> 60	216/208/28	201/141/39	1.437(1.072-1.926)	0.015	0.708/0.292	0.713/0.287	0.98(0.79-1.21)	0.835

*: Adjusted by gender, BMI, TC, TG, Cr, HDL-C, LDL-C, Glu, smoking, drinking.

**Table 5:** Analysis of interaction of gene and age for EH.

Genotype		Age group (years)	β	Standard error	P	OR (95% CI)
Rs3754734	TG/GG	< 50	-	-	-	1.00
(TG/GG vs. TT)	TG/GG	50-60 vs. < 50	0.252	0.203	0.215	1.286(0.864-1.916)
	TG/GG	> 60 vs. < 50	0.564	0.204	0.006	1.758 (1.180-2.620)
rs2011616	GA/AA	< 50	-	-	-	1.00
(GA/AA vs. GG)	GA/AA	50-60 vs. < 50	0.315	0.202	0.118	1.371(0.923-2.035)
	GA/AA	> 60 vs. < 50	0.643	0.202	0.001	1.903(1.281-2.825)

*: Adjusted by gender, BMI, TC, TG, Cr, HDL-C, LDL-C, Glu, smoking, drinking.

Pairwise linkage disequilibrium (LD) analysis between the SNPs was measured by genetic program with R software and the value of D' equal to 0.827 between rs3754734 and rs2011616. To further investigate the effect of haplotype and interaction in stage 2, haplotypes were constructed by the two SNPs arranged in the order of rs3754734 and rs2011616. The frequencies of haplotype hap1(TG), hap2(GA), hap3(GG) and hap4(TA) were 0.613 (base haplotype), 0.265, 0.088 and 0.033 respectively (except 0.001 for rare haplotype constructed by not typed genotype). Haplo.glm model was used with R software and the results showed that there weren't any haplotypes associated with EH but the interaction of hap2 and age-group found to be significant after being adjusted for the covariates above, OR was 1.220(1.031-1.444), P value was 0.020.

Additionally, the comparisons of quantitative trait for blood pressure were processed among genotypes. Normal test indicated that blood pressure presented approximate normal distribution. The results of one-way ANOVA showed that the differences of SBP and DBP in treatment (n = 448) [24] and DBP in non-treatment groups (n = 2115) were significant between genotypes of rs3754734 (table 6). But no significant alterations of SBP or DBP were found between the genotypes of rs2011616.

**Table 6:** Comparisons of quantitative traits of blood pressure between genotypes of rs3754734 and 2011616 by treatment stratification

	rs3754734			rs2011616		
		
Group	TT	TG	GG	GG	GA	AA
Treatment (n)	196	200	52	225	190	33
SBP (mmHg)	174.03 ± 25.73	176.24 ± 20.72	184.20 ± 22.41*	177.55 ± 25.95	174.78 ± 20.62	176.08 ± 18.89
DBP (mmHg)	102.45 ± 10.75	103.64 ± 9.55	105.93 ± 10.50*	103.53 ± 10.94	103.11 ± 9.49	104.87 ± 9.57
Non-treatment (n)	881	960	274	1025	906	186
SBP (mmHg)	126.91 ± 21.15	128.65 ± 24.26	128.42 ± 20.86	127.60 ± 21.67	128.38 ± 22.52	127.54 ± 27.56
DBP (mmHg)	79.72 ± 12.23	80.19 ± 12.87	81.66 ± 12.37*	80.37 ± 12.48	79.98 ± 12.77	80.57 ± 12.08

P > 0.05 for all test of homogeneity of variances

*P < 0.05 for linear term test

## Discussion

Emilin1 gene was located in 2p23.3-p23.2. As a member of EMILINs, which are a new family of glycoprotein's of the extracellular matrix [25], Emilin1 localized at the interface between amorphous elastin and microfibril and played an important role in embryonic development [26], blood pressure homeostasis by linking TGF-β maturation [2,7], elastogenesis and vascular cell defects [9]. The gC1q domain of Emilin1 can form relatively stable and compact homotrimers, and then be followed by a multimeric assembly of disulfide-bonded protomers. The transcription of Emilin1 in different tissues is probably achieved through combinatorial cooperation between various regions, rather than being dependent on a single cis-activating region specific for each tissue [27]. Emilin1 code for a secreted proteins whose hallmark is the presence of a cysteine-rich motif defined as EMI domain [28] that followed by a coiled-coil, a short collagenous domain, and a C1q-like repeat at the C terminus which has been shown that the C-terminal domain is involved in the oligomerization of Emilin1, cell adhesion and modulating growth factors' activity [29].

Zanetti et al. [2,9] proved that an unprecedented link between TGF-β signaling and arterial hypertension was based on the analysis of mice mutant for Emilin1, which genetic inactivation would induce TGF-β signaling up-regulation in the vascular wall and further cause blood pressure increasing. Therefore, the findings reported stimulate further study to replicate in human and we designed the present study to explore the association of Emilin1 gene with EH in a northern Han Chinese population.

With the purpose of rationality and availabiliy in research, we mainly considered the following approaches. First, we focused on a segment located from 5 kb upstream of Emilin1 gene to 1 kb downstream and mainly aimed at SNPs with a minimum MAF ≥ 0.05. We searched HapMap database of CHB recently published and found three SNPs with available MAF. We tested all those three SNPs in present study rather than general haplotype-tagging methods for selecting SNPs as Bhatti described [12]. Second, we applied a two-stage study to test the association of Emilin1 gene with EH. As well known, two-stage study provides a practical cost-effective strategy for association studies and full set of SNPs genotyping is performed in the first stage in a fraction (allocate 1/3 to 1/2) of the total number of samples for screening [21,22]. In addition, the hypertensive cases with higher BP level in stage 1 were likely to be enriched genetic susceptibility and might increase power to test the association of Emilin1 gene with EH [16].

Blood pressure has long been known to be a complex trait influenced by both genetic and environmental factors [30]. A large number of genes are potentially involved in blood pressure regulation and even the susceptibility to hypertension, and the effects of these genes may be modulated by age [31,32]. Age-genetic effects for complex traits, such as hypertension [33-35], can prevent replications if the age-varying character of an association is not taken into account in the selection of both the replication samples and the statistical analysis strategy [34]. Because age is a complex biological construction, it may serve as a surrogate for a variety of interacting covariates and which may not only enhance gene discovery, but also render findings from across studies more comparable to each other [32]. As well known, for subjects over 50 years of age, SBP and DBP increase exponentially with age [36], so we divided age into < 50, 50-60 and ≥ 60 years groups and this age stratification may help estimate the ages at which subjects should be studied to maximize the expression of the genetic effect and increase the power of association studies for various phenotypes [31,34,36].

In the present study, our results showed that two SNPs, rs3754734 and rs2011616 had significant association with EH in stage 1. But association analyses for replicated authentication in stage 2 showed that the significant associations hadn't been repeated directly. Some reasons as follow probably contribute to this disaccord. Firstly, the blood pressure levels of cases in sample 2 were lower than sample 1 so that the association strength was decrease in joint sample and hardly detected. Secondly, false positive association presented in stage 1 was rooted in potential differentiation for covariates or population structure in the two samples. Thirdly, genetic heterozygosity arose from the inner of research population might devote different susceptibility to EH in subgroups. Also, phenocopy for other disease often falsified results in association study. Finally, information bias including measurement bias, recall bias, misclassification, etc. often distort results for epidemiological association studies. Further age-stratified analysis and interaction detection showed that the variations of Emilin1 gene might influence the susceptibility to EH via age modulating way. Accordance to the biologically plausibility described as reported above, it would be helpful to interpret that whether potential biological roles of rs3754734 on transcriptional control and rs2011616 on TF binding sites would affect the susceptibility alteration to EH or blood pressure homeostasis in human.

In conclusion, our finding suggests age modulated association between the genetic variation of Emilin1 gene and blood pressure ascending and that would stimulate further investigation of the role of Emilin1 gene in vascular development and blood pressure homeostasis. Meanwhile, it is worthwhile to find the biological function of Emlin1 in signal transforming and regulating pathway involved in blood advance[37]. Additionally, the Emlin1 sequencing or searching SNPs data of other races would provide more SNPs to select except for following interests in SNPs with MAF < 0.05 or other variants farther away from Emilin1 gene to provide rationality support [38,39].

## Conclusion

Our findings support that the Emilin1 gene had an age modulated interaction on the risk to EH and further replications in other populations and functional studies should be warranted.

## Abbreviations

ANOVA: Analysis of (variance); (BMI): Body mass index; (χ^2^): Chi-square; (Cr): Creatinine; (DBP): Diastolic BP; (EDTA): Ethylenediamine tetraacetic acid; (ECM): Elastic extracellular matrix; (EH): Essential hypertension; (Glu): Glucose; (CHB): Han Chinese in Beijing, China; (HWE): Hardy-Weinberg equilibrium; (HDL-C): High density lipoprotein cholesterol; (LD): Linkage disequilibrium; (LDL-C): Low density lipoprotein cholesterol; (MAF): Minor allele frequency; (ORs): Odds ratio; (PCR/RFLP): Polymerase chain reaction/restriction fragment length polymorphism; (P): Probability; (SNPs): Single nucleotide polymorphisms; (SBP): Systolic BP; (Sp1): Stimulating protein 1; (TF): Transcription factor; (TG): Triglycerides; (TC): Total cholesterol; (InterASIA): The International Collaborative Study of Cardiovascular Disease in Asia; (TGF): Transforming growth factor.

## Competing interests

The authors declare that they have no competing interests.

## Authors' contributions

CS designed the study and carried out the genotyping work, statistical analyzing and drafted the manuscript. YL, XL and LYW participated in genotyping. XFL and QZ participated in analyzing data and XFL participated in drafting and revising the manuscript. LPH and SFC participated in the analyses of bioinformatics. JFH and DFG are the principal investigators of the study and DFG helped in study design and supervised all process of the study. All authors have read and approved the final manuscript.

## Pre-publication history

The pre-publication history for this paper can be accessed here:

http://www.biomedcentral.com/1471-2350/10/118/prepub

## References

[B1] Staessen JA Wang J Bianchi G Birkenhager WH Essential hypertension Lancet 2003 361 1629 1641 10.1016/S0140-6736(03)13302-8 12747893

[B2] Zacchigna L Vecchione C Notte A Cordenonsi M Dupont S Maretto S Cifelli G Ferrari A Maffei A Fabbro C Emilin1 links TGF-beta maturation to blood pressure homeostasis Cell 2006 124 929 942 10.1016/j.cell.2005.12.035 16530041

[B3] Lee DS Massaro JM Wang TJ Kannel WB Benjamin EJ Kenchaiah S Levy D D'Agostino RB Sr Vasan RS Antecedent blood pressure, body mass index, and the risk of incident heart failure in later life Hypertension 2007 50 869 876 10.1161/HYPERTENSIONAHA.107.095380 17893376

[B4] Arribas SM Hinek A Gonzalez MC Elastic fibres and vascular structure in hypertension Pharmacol Ther 2006 111 771 791 10.1016/j.pharmthera.2005.12.003 16488477

[B5] Argano C Duro G Corrao S Di Chiara T Nuzzo D Colomba D Scaglione R Licata G Transforming growth factor beta1 T29C gene polymorphism and hypertension: relationship with cardiovascular and renal damage Blood Press 2008 17 220 226 10.1080/08037050802431416 18821144

[B6] Schiffrin EL Remodeling of resistance arteries in essential hypertension and effects of antihypertensive treatment Am J Hypertens 2004 17 1192 1200 10.1016/j.amjhyper.2004.05.023 15607629

[B7] Raman M Cobb MH TGF-beta regulation by Emilin1: new links in the etiology of hypertension Cell 2006 124 893 895 10.1016/j.cell.2006.02.031 16530037

[B8] August P Suthanthiran M Transforming growth factor beta signaling, vascular remodeling, and hypertension N Engl J Med 2006 354 2721 2723 10.1056/NEJMcibr062143 16790709

[B9] Zanetti M Braghetta P Sabatelli P Mura I Doliana R Colombatti A Volpin D Bonaldo P Bressan GM EMILIN-1 deficiency induces elastogenesis and vascular cell defects Mol Cell Biol 2004 24 638 650 10.1128/MCB.24.2.638-650.2004 14701737 PMC343785

[B10] Bressan GM Daga-Gordini D Colombatti A Castellani I Marigo V Volpin D Emilin, a component of elastic fibers preferentially located at the elastin-microfibrils interface J Cell Biol 1993 121 201 212 10.1083/jcb.121.1.201 8458869 PMC2119774

[B11] Thomas D Xie R Gebregziabher M Two-Stage sampling designs for gene association studies Genet Epidemiol 2004 27 401 414 10.1002/gepi.20047 15543639

[B12] Gu D Reynolds K Wu X Chen J Duan X Muntner P Huang G Reynolds RF Su S Whelton PK He J Prevalence, awareness, treatment, and control of hypertension in china Hypertension 2002 40 920 927 10.1161/01.HYP.0000040263.94619.D5 12468580

[B13] Gu D Reynolds K Wu X Chen J Duan X Reynolds RF Whelton PK He J Prevalence of the metabolic syndrome and overweight among adults in China Lancet 2005 365 1398 1405 10.1016/S0140-6736(05)66375-1 15836888

[B14] Perloff D Grim C Flack J Frohlich ED Hill M McDonald M Morgenstern BZ Human blood pressure determination by sphygmomanometry Circulation 1993 88 2460 2470 8222141 10.1161/01.cir.88.5.2460

[B15] Lu X Zhao W Huang J Li H Yang W Wang L Huang W Chen S Gu D Common variation in KLKB1 and essential hypertension risk: tagging-SNP haplotype analysis in a case-control study Hum Genet 2007 121 327 335 10.1007/s00439-007-0340-4 17318641

[B16] Zhao Q Fan Z He J Chen S Li H Zhang P Wang L Hu D Huang J Qiang B Gu D Renalase gene is a novel susceptibility gene for essential hypertension: a two-stage association study in northern Han Chinese population J Mol Med 2007 85 877 885 10.1007/s00109-006-0151-4 17216203

[B17] Sharma P Fatibene J Ferraro F Jia H Monteith S Brown C Clayton D O'Shaughnessy K Brown MJ A genome-wide search for susceptibility loci to human essential hypertension Hypertension 2000 35 1291 1296 10856279 10.1161/01.hyp.35.6.1291

[B18] Gu D Su S Ge D Chen S Huang J Li B Chen R Qiang B Association study with 33 single-nucleotide polymorphisms in 11 candidate genes for hypertension in Chinese Hypertension 2006 47 1147 1154 10.1161/01.HYP.0000219041.66702.45 16636198

[B19] Yuan HY Chiou JJ Tseng WH Liu CH Liu CK Lin YJ Wang HH Yao A Chen YT Hsu CN FASTSNP: an always up-to-date and extendable service for SNP function analysis and prioritization Nucleic Acids Res 2006 34 W635 641 10.1093/nar/gkl236 16845089 PMC1538865

[B20] Guo SW Thompson EA Performing the exact test of Hardy-Weinberg proportion for multiple alleles Biometrics 1992 48 361 372 10.2307/2532296 1637966

[B21] Skol AD Scott LJ Abecasis GR Boehnke M Joint analysis is more efficient than replication-based analysis for two-stage genome-wide association studies Nat Genet 2006 38 209 213 10.1038/ng1706 16415888

[B22] Zuo Y Zou G Wang J Zhao H Liang H Optimal two-stage design for case-control association analysis incorporating genotyping errors Ann Hum Genet 2008 72 375 387 10.1111/j.1469-1809.2007.00419.x 18215207 PMC2836813

[B23] Lake SL Lyon H Tantisira K Silverman EK Weiss ST Laird NM Schaid DJ Estimation and tests of haplotype-environment interaction when linkage phase is ambiguous Hum Hered 2003 55 56 65 10.1159/000071811 12890927

[B24] Levy BI Ambrosio G Pries AR Struijker-Boudier HA Microcirculation in hypertension: a new target for treatment? Circulation 2001 104 735 740 10.1161/hc3101.091158 11489784

[B25] Doliana R Mongiat M Bucciotti F Giacomello E Deutzmann R Volpin D Bressan GM Colombatti A EMILIN, a component of the elastic fiber and a new member of the C1q/tumor necrosis factor superfamily of proteins J Biol Chem 1999 274 16773 16781 10.1074/jbc.274.24.16773 10358019

[B26] Beck S Le Good JA Guzman M Ben Haim N Roy K Beermann F Constam DB Extraembryonic proteases regulate Nodal signalling during gastrulation Nat Cell Biol 2002 4 981 985 10.1038/ncb890 12447384

[B27] Fabbro C de Gemmis P Braghetta P Colombatti A Volpin D Bonaldo P Bressan GM Analysis of regulatory regions of Emilin1 gene and their combinatorial contribution to tissue-specific transcription J Biol Chem 2005 280 15749 15760 10.1074/jbc.M412548200 15705587

[B28] Braghetta P Ferrari A De Gemmis P Zanetti M Volpin D Bonaldo P Bressan GM Overlapping, complementary and site-specific expression pattern of genes of the EMILIN/Multimerin family Matrix Biol 2004 22 549 556 10.1016/j.matbio.2003.10.005 14996434

[B29] Mongiat M Mungiguerra G Bot S Mucignat MT Giacomello E Doliana R Colombatti A Self-assembly and supramolecular organization of EMILIN J Biol Chem 2000 275 25471 25480 10.1074/jbc.M001426200 10821830

[B30] Hamet P Pausova Z Adarichev V Adaricheva K Tremblay J Hypertension: genes and environment J Hypertens 1998 16 397 418 10.1097/00004872-199816040-00001 9797185

[B31] Bao X Mills PJ Rana BK Dimsdale JE Schork NJ Smith DW Rao F Milic M O'Connor DT Ziegler MG Interactive effects of common beta2-adrenoceptor haplotypes and age on susceptibility to hypertension and receptor function Hypertension 2005 46 301 307 10.1161/01.HYP.0000175842.19266.95 16027244

[B32] Shi G Gu CC Kraja AT Arnett DK Myers RH Pankow JS Hunt SC Rao DC Genetic effect on blood pressure is modulated by age: the Hypertension Genetic Epidemiology Network Study Hypertension 2009 53 35 41 10.1161/HYPERTENSIONAHA.108.120071 19029486 PMC2633773

[B33] Percy CJ Brown L Power DA Johnson DW Gobe GC Obesity and hypertension have differing oxidant handling molecular pathways in age-related chronic kidney disease Mech Ageing Dev 2009 130 129 138 10.1016/j.mad.2008.10.003 19041334

[B34] Lasky-Su J Lyon HN Emilsson V Heid IM Molony C Raby BA Lazarus R Klanderman B Soto-Quiros ME Avila L On the replication of genetic associations: timing can be everything! Am J Hum Genet 2008 82 849 858 10.1016/j.ajhg.2008.01.018 18387595 PMC2427263

[B35] Lunetta KL D'Agostino RB Sr Karasik D Benjamin EJ Guo CY Govindaraju R Kiel DP Kelly-Hayes M Massaro JM Pencina MJ Genetic correlates of longevity and selected age-related phenotypes: a genome-wide association study in the Framingham Study BMC Med Genet 2007 8 Suppl 1 S13 10.1186/1471-2350-8-S1-S13 17903295 PMC1995604

[B36] Safar ME Lajemi M Rudnichi A Asmar R Benetos A Angiotensin-converting enzyme D/I gene polymorphism and age-related changes in pulse pressure in subjects with hypertension Arterioscler Thromb Vasc Biol 2004 24 782 786 10.1161/01.ATV.0000119354.41615.33 14751812

[B37] Dao HH Essalihi R Bouvet C Moreau P Evolution and modulation of age-related medial elastocalcinosis: impact on large artery stiffness and isolated systolic hypertension Cardiovasc Res 2005 66 307 317 10.1016/j.cardiores.2005.01.012 15820199

[B38] Bhatti P Church DM Rutter JL Struewing JP Sigurdson AJ Candidate single nucleotide polymorphism selection using publicly available tools: a guide for epidemiologists Am J Epidemiol 2006 164 794 804 10.1093/aje/kwj269 16923772

[B39] Barrett JC Fry B Maller J Daly MJ Haploview: analysis and visualization of LD and haplotype maps Bioinformatics 2005 21 263 265 10.1093/bioinformatics/bth457 15297300

